# Knowledge of Dental Ethics and Jurisprudence Among Dental Practitioners in Pune: A Questionnaire Survey

**DOI:** 10.7759/cureus.31503

**Published:** 2022-11-14

**Authors:** Shrikanth Muralidharan, Gowri Pendyala, Dhananjay Gandhage, Saraswati Sachan

**Affiliations:** 1 Dentistry, Private Practitioner, Pune, IND; 2 Periodontics, Rural Dental College, Pravara Institute of Medical Sciences, Loni, IND; 3 Prosthodontics, Dr. D. Y. Patil Dental College & Hospital, Dr. D. Y. Patil Vidyapeeth, Pune, IND; 4 Conservative Dentistry and Endodontics, Sardar Patel Post Graduate Institute of Dental and Medical Sciences, Lucknow, IND

**Keywords:** professional behaviour, pune, jurisprudence, ethics, dentist

## Abstract

Objective

Dental ethics is a moral obligation that solicits professional behaviour imposed by the members of the dental profession. In addition, a set of legal regulations by each state's legislature describing the legal limitations and regulations related to dentistry, dental hygiene, and dental assisting is a part of dental jurisprudence. The present study aims to evaluate the knowledge regarding ethics and medical law for the practice among private practitioners in Pune, India.

Materials and methods

This was a cross-sectional study. The snowball sampling method (a randomization method) was used to contact all the registered practitioners for the study. A total of 250 dentists agreed to provide written informed consent and participate in the study. The collected data were entered into Microsoft Excel 2013 sheet (Microsoft Corporation, Redmond, WA) and cleaned, and statistical analysis was carried out using Epi Info software (CDC, Atlanta, GA). All p-values below 0.05 were considered to be statistically significant.

Results

A total of 250 dentists participated in the present study. Female postgraduate dentists had lesser knowledge of abbreviations used on dental boards. Master of Dental Surgery (MDS) dentists had more knowledge regarding dental jurisprudence than Bachelor of Dental Surgery (BDS) practitioners.

Conclusions

The present study highlighted the lack of awareness regarding dental ethics and jurisprudence among dental practitioners. It underlines the need for more education programs and curriculum changes with a focus on ethics, jurisprudence, and legal matters pertaining to clinical practice.

## Introduction

The ethical dilemma has been a part of the healthcare profession. Healthcare workers have been confronted with questions regarding medical ethics since medicine has been practised [1]. Nowadays, its magnitude has changed, including issues that every practitioner needs to be thoughtful about. The knowledge of medical ethics and the taking of moral positions are essential parts of everyday clinical practice. In borderline situations, such as deciding the type of intervention, autonomy and responsibility for the treatment outcomes must reflect the practitioners' moral values. Apart from written laws, professional, ethical principles underpin a capacity for critical judgement to ensure treatment decisions are well-grounded and appropriate [2]. A Bavarian study reported that doctors who have been practising for longer believe that patients have little comprehension of the consequences of therapeutic decisions. It could be related to the limited role ascribed to patient autonomy [2]. Ethical education has been included in the training curriculum of health professionals in many countries, and there has been a growth in the number of ethics specialists and committees. However, still, public complaints have been increasing in recent times. Few health professionals were exposed to a detailed training process; nevertheless, they are still expected to know about these aspects and apply them practically [3-5]. Dental ethics is a moral obligation that solicits professional behaviour imposed by the members of the dental profession. The dental regulations (Code of Ethics) were laid down by the Dental Council of India (DCI) in 1976 and later revised in 2014. Every registered dentist must understand their responsibilities and abide by them the same [6]. A set of legal regulations by each state's legislature describing the legal limitations and regulations related to dentistry, dental hygiene, and dental assisting is a part of dental jurisprudence. The Dentists Act of 1948 is directly concerned with the statutory regulation of the dental profession. The Consumer Protection Act (COPRA), Indian Contracts Act, and Indian Penal Code also govern dental practice. Dentists must know in detail about these acts to have a legally and medically competent practice [7]. The present study aims to evaluate the knowledge regarding ethics and medical law for the practice among private practitioners in Pune, India.

## Materials and methods

Ethical clearance was obtained from the Institutional Review Board of Rural Dental College (number: IEC/MARDC/2021) before the start of the study. The study was cross-sectional. It was carried out from January 2021 to April 2021. We used the snowball sampling method wherein the participants were known to the authors and referred to other academicians across different colleges in India for the study. The questionnaire used was already validated in a previous study done in South India [7]. Of the 480 dentists contacted, 300 dentists agreed to provide online digital informed consent and participate in the study. Of these 300, only 250 returned the filled-in online questionnaires. Incomplete questionnaires were not included in the final study. The data collected were entered into Microsoft Excel 2013 sheet (Microsoft Corporation, Redmond, WA) and cleaned, and statistical analysis was carried out using Epi Info software (CDC, Atlanta, GA). All chi-square tests were performed to evaluate the differences between the participating dentists' knowledge, qualification, and gender. All p-values below 0.05 were considered to be statistically significant.

## Results

A total of 250 dentists participated in the present study, of which 75 were Bachelor of Dental Surgery (BDS; general practitioners) and 175 were Master of Dental Surgery (MDS) specialists. The distribution of dentists based on their correct knowledge of the Dentist Act and the Dental Code of Ethics is shown in Table 1.

**Table 1:** Distribution of the study participants based on their knowledge regarding the Dentist Act and Dental Code of Ethics BDS: Bachelor of Dental Surgery; MDS: Master of Dental Surgery; n: number of participants.

Qualification	BDS		MDS		Total n (%)	P-values
Gender	Male, n (%)	Female, n (%)	Male, n (%)	Female, n (%)		
Year the Dentist Act was passed	14 (36.8)	20 (54.05)	41 (51.2)	54 (56.8)	129 (51.6)	<0.05
Year the Dental Code of Ethics was given	8 (21.05)	10 (27.02)	20 (25)	28 (29.5)	66 (26.4)	<0.05
Revision of the Code of Ethics	5 (13.1)	5 (13.7)	10 (12.5)	13 (13.7)	33 (13.2)	<0.05
Number of principles in the Code of Ethics	10 (26.3)	10 (27.02)	29 (36.3)	30 (31.8)	79 (31.6)	<0.05

Most female MDS degree holders had more excellent knowledge than male and female BDS practitioners, and this difference was statistically significant. Female MDS dentists had a lesser understanding of abbreviations used on the dental boards; there was greater awareness amongst all the practitioners about the revised DCI norms of 2014 (Table 2).

**Table 2:** Distribution of dentists based on their knowledge regarding unethical practices BDS: Bachelor of Dental Surgery; MDS: Master of Dental Surgery.

Principles	BDS		MDS			
	Male, n (%)	Female, n (%)	Male, n (%)	Female, n (%)	Total, n (%)	P-value
Dentists cannot use abbreviations of memberships in associations or organizations as a suffix to their names	20 (52.6)	19 (51.3)	42 (52.5)	45 (47.4)	126 (50.4)	<0.05
It is unethical for a dental surgeon to refuse treatment because the patient is HIV positive or suffering from any other contagious disease	30 (78.9)	30 (81.1)	65 (81.2)	80 (84.2)	205 (82)	<0.05
According to the revised code, it is not unethical to advertise a dental clinic	15 (39.5)	16 (43.2)	33 (41.2)	42 (44.2)	106 (42.4)	<0.05
According to the revised code, it is unethical to use a dentist’s name in commercial products like toothpaste and toothbrush	19 (50)	20 (54.1)	41 (51.2)	50 (52.6)	130 (52)	<0.05
According to the revised code, it is unethical to affix a signboard in a chemist's shop or in other places where the dentist does not reside or work	30 (85.7)	32 (86.5)	70 (87.5)	90 (94.7)	222 (88.8)	<0.05
It is not unethical for a dental surgeon to supply or sell drugs in his clinic	20 (52.6)	18 (48.6)	40 (50)	50 (52.6)	128 (51.2)	<0.05
According to the revised code, it is unethical for a dental surgeon to receive gifts from pharmaceutical companies	17 (44.7)	23 (62.1)	52 (65)	60 (63.6)	152 (60.8)	<0.05
Total	38	37	80	95	250	<0.05

According to the results obtained from evaluating the questionnaires, MDS dentists had significantly greater knowledge regarding dental jurisprudence than BDS practitioners (Table 3).

**Table 3:** Distribution of dentists based on their knowledge regarding dental jurisprudence BDS: Bachelor of Dental Surgery; MDS: Master of Dental Surgery.

Dental jurisprudence	BDS		MDS			
	Male, n (%)	Female, n (%)	Male, n (%)	Female, n (%)	Total, n (%)	P-value
It is not necessary to obtain informed consent for clinical examination and routine radiography	19 (50)	19 (51.3)	48 (60)	57 (60)	143 (57.2)	<0.05
Assent is taken for children above 12 years and below 18 years	16 (42.1)	17 (45.9)	43 (53.7)	51 (53.7)	127 (50.8)	<0.05
Records need to be maintained for at least 5 years	13 (34.2)	14 (37.8)	30 (37.5)	40 (42.1)	97 (38.8)	<0.05
Consumer Protection Act does not extend to free hospital-based treatments	20 (52.6)	20 (54.05)	45 (56.25)	56 (58.9)	141 (56.4)	<0.05
Total	38	37	80	95	250	<0.05

## Discussion

The present study was the first to highlight the knowledge related to dental ethics and jurisprudence among practitioners in Pune. Only 26.4% knew about the year the code of ethics was given; the number was even smaller with respect to the year of revision of those codes. More than a quarter of the participants had information about the principles of dental ethics. Compared to the study done among practitioners in Chennai city where 19% knew that the dentists regulation (Code of Ethics) came into force in 1976, and 32% were aware that the code was revised, more dentists knew about this in our study [7]. Approximately 50% of the dentists in the present study were unaware that abbreviations apart from their degrees cannot be mentioned on the signboards of the dental clinics, similar to the study's findings in Chennai [8]. Only 42% knew that it is not unethical to advertise one's clinic as per the revised rules, similar to the other study [9]. According to another study in Bangalore, younger dentists were more open to advertisements than senior dentists [10,11]. Another study argued that this either reflects a drop in ethical standards or perhaps the need to utilize advertisement as a means to reach out to people who are potential customers [11,12]. Santos Pacheco et al. stated that among 529 cases, 39.7% of dentists were supposedly related to illicit advertising. The dental code of conduct permits advertisement about hospital inauguration, shifting, and services provided at the hospital, but it has to have a prescribed size and format. This code is underserving in today's modern era [13]. Of the dentists in the present study, 82% said that it is unethical to refuse treatment to HIV-positive patients, which was higher than the study findings of two other previous studies [7,8]. In the present study, 51.2% said it is unethical to sell drugs at their dental clinics, similar to Chennai's study [7]. A higher percentage of dentists in the present study knew that routine check-up and radiography does not require specific written consent, which was higher than the report by Kesavan et al. (31%) and lesser than that reported by Janakiram and Gardens (77%) [7,9]. Consent is not just a safeguard for the dentists but can also build trust between the patients and the dentists since the patients are empowered to make informed decisions on their health [14,15]. Singh et al. reported that awareness of COPRA was much higher among medical practitioners than dentists [10]. This difference could be attributed to the number of negligence cases the medical professional witness. Milgrom et al. observed that the most significant damage was from oral surgery and prosthodontics [14]. More than half of the dentists in the present study knew that free treatments are exempted from the Consumer Protection Act. It was higher than the report by Kesavan et al. (35%) and much lesser than the study by Singh et al. (90%) [7,10]. Overall, dental practitioners had a dearth of knowledge concerning dental ethics and jurisprudence. Our study did not obtain information on years of practice and institution affiliation. Previous study data, though, highlights the poor knowledge among dental college-associated practitioners [7]. Dental practice is a challenging task with several hurdles, including legal ones. Lack of awareness could cause lasting damage to both dentists and patients. Research has shown that physicians, dentists, and nurses, even though they appreciate the need for ethics, poor knowledge leads to violation of these principles [16-18]. With dental patients having an armchair of knowledge, it is even more challenging to keep up with work's legal and professional aspects. It is also up to the professional bodies to try coming up with continuing dental education (CDE) programs to spread awareness related to the same.

The study's main limitation was that it was based on subjective assessment. There is no actual judgement of their attitudes and practices as well. As in this study, the professionals associated with dental colleges were included through random cluster sampling, and one major disadvantage is "recall bias", where the respondent's older experiences influence his/her memory.

## Conclusions

The present study highlighted the lack of awareness regarding dental ethics and jurisprudence among dental practitioners. It underlines the fact for more ease of accessibility of information to dentists, CDE programs, more extensive nationwide surveys, curriculum changes with more focus on ethics, jurisprudence, and legal matters pertaining to clinical practice, and also interest from the side of the clinicians to be aware and updated with newer and reformed rules and regulations are the need of the hour.
